# Doxorubicin aqueous systems at low concentrations: Interconnection between self-organization, fluorescent and physicochemical properties, and action on hydrobionts

**DOI:** 10.3389/fchem.2022.1063278

**Published:** 2022-12-01

**Authors:** Irina Ryzhkina, Lyaisan Murtazina, Larisa Kostina, Irina Dokuchaeva, Svetlana Sergeeva, Kristina Meleshenko, Maxim Shevelev, Andrew Petrov

**Affiliations:** ^1^ Arbuzov Institute of Organic and Physical Chemistry, FRC Kazan Scientific Center, Russian Academy of Sciences, Kazan, Russia; ^2^ Institute for Problems of Ecology and Mineral Wealth Use of Tatarstan Academy of Sciences, Kazan, Russia

**Keywords:** self-organization, diluted dispersed systems, doxorubicin, hydrobionts, fluorescence

## Abstract

Doxorubicin (Dox) is a highly effective cytostatic antibiotic that exhibits activity against a wide range of malignant neoplasms and is often used as the basis of various anti-tumor compositions. However, the use of Dox in therapeutic doses is associated with high systemic toxicity, which makes it urgent to find ways to reduce therapeutic concentrations, which is necessary primarily to minimize the side effects on the patient’s body, as well as to reduce the harmful effects on aquatic ecosystems, commonly polluted by toxic pharmaceuticals. Studying the self-organization, physicochemical and spectral patterns, and their relation to bioeffects of Dox solutions in the range of low concentrations can reveal useful insights into the unknown effects of Dox as a cytostatic and potential pollutant of ecosystems. The self-organization in solutions and on substrates, physicochemical and spectral properties, and action of Dox solutions on hydrobionts were studied in the range of calculated concentrations from 1·10^−20^ to 1·10^−4^ M by methods of dynamic and electrophoretic light scattering (DLS and ELS), scanning electron microscopy (SEM), scanning probe microscopy (SPM), fluorescence spectroscopy, UV absorption spectroscopy, conductometry, tensiometry, pH-metry. Certified techniques for monitoring the toxicity of natural water and wastewater were used to establish the interconnection between these phenomena. It was shown that aqueous solutions of Dox are dispersed systems which rearrange their dispersed phase measuring hundreds of nm in size (nanoassociates) at dilution, followed by concerted changes in nanoassociates’ parameters (size and ζ-potential) and properties of systems, as well as their bioassay results. SPM and SEM results confirm and complement the DLS and ELS data indicating the existence of nanoassociates in dilute Dox solutions.

## Introduction

Establishment of the structure, reactivity and physicochemical patterns of diluted aqueous solutions of biologically active substances (BAS) is closely related to the task of scientific substantiation of the features of their bioeffects, such as nonmonotonic concentration dependences, the presence of “silent zones” where a biosystem is almost insensitive to a drug, a change in bioeffect sign as the solution is being diluted, etc. It is important to solve the problem of structural and biological effects of diluted aqueous solutions to create effective and safe drugs for the treatment of humans and animals, improve the environmental situation, increase crop yields (Mark and Edward, 2009; Santos et al., 2010; Shimanovsky et al., 2010).

A recently developed physicochemical approach to the study of diluted aqueous solutions of BAS explains significant non-monotonic changes in the properties and bioeffects of such solutions in terms of self-organization of dispersed systems. Examples of a wide range of different BAS were used to establish that dilute aqueous solutions below a threshold concentration (*c*_thr_) are open self-organized disperse systems capable of formation and rearrangement of the disperse phase (domain-nanoassociate, nanoassociate-nanoassociate), whose size and zeta-potential change non-monotonically with dilution, which is reflected in a coherent change in the reactivity, catalytic activity, physicochemical, spectral, and biological properties of the systems, most pronounced at critical concentrations (Ryzhkina et al., 2009; Ryzhkina et al., 2011; Konovalov and Ryzhkina, 2014; Konovalov A. et al., 2015; Konovalov D. A. et al., 2015; Ryzhkina et al., 2017; Ryzhkina et al., 2020; Ryzhkina et al., 2021a; Ryzhkina et al., 2021b; Ryzhkina et al., 2021c). Obtained results allow for the conclusion that the transformation of nanoassociates upon systems’ dilution underlies the features of the nonmonotonic pharmacological profiles of the diluted solutions of BAS (Konovalov and Ryzhkina, 2014).

It was shown that nanoassociates are discrete water-molecular fractal objects of hundreds of nm in size, formed in individual and multicomponent diluted aqueous and water-organic systems of BAS, having an electrically charged interface (zeta-potential ranging from −2 to −20 mV). The conditions for the formation of nanoassociates area certain structure of the dissolved substance, use of a special solution preparation procedure, the presence of background electromagnetic fields (Konovalov and Ryzhkina, 2014; Lunev et al., 2014; Gall and Gall, 2015; Konovalov A. et al., 2015; Konovalov D. A. et al., 2015; Pershin et al., 2016; Ryzhkina et al., 2017; Ryzhkina et al., 2020). Similar to soft nanomaterials such as nanogels (Gonsalves et al., 2007; Kabanov and Vinogradov, 2009), nanoassociates are signal-sensitive structures that respond to external factors - changes in pH, temperature, and the frequency and amplitude of electromagnetic fields. The formation and rearrangement of nanoassociates is connected with the appearance of absorption bands in the 200–280 nm range and emission bands in the 300–450 nm spectral range in UV spectra, reflecting their intrinsic ability to absorb and emit energy in the UV region in certain concentration and temperature ranges (Ryzhkina et al., 2021a; Ryzhkina et al., 2021b; Ryzhkina et al., 2021c).

To deepen the understanding of the nature of the detected phenomena and their connection to the real problems of pharmacology and medicine, this work studies an aqueous solution of doxorubicin hydrochloride (Dox). Doxorubicin is a highly effective cytostatic antibiotic that exhibits activity against a wide range of malignant neoplasms. Dox is often used both in monotherapy of tumors and as a base of various antitumor compositions (Rivankar, 2014). However, the use of Dox in therapeutic concentrations (about 1.10^−3^ M) is associated with high systemic toxicity, which makes the search for ways to reduce therapeutic doses very relevant, primarily to minimize the side effects on the patient body (Rivankar, 2014; Guven et al., 2018; Prasanna et al., 2020), as well as to reduce the harmful effects on aquatic ecosystems, widely polluted by toxic pharmaceuticals (Santos et al., 2010; Ryzhkina et al., 2020; Chow et al., 2021; Wilkinson et al., 2022), which also include Dox as well (Lenz et al., 2007; Mahnik et al., 2007).

A study of the possible use of diluted (1.10^−5^–1.10^−20^ M) Dox solutions in the chemotherapy of tumors has shown promising prospects for their use in clinical practice. When studying the effects of diluted Dox solutions on the development of animal tumor models, a non-monotonic character of the concentration dependencies of the antitumor effect was found, which in certain concentration ranges is comparable with the inhibitory activity of the drug in therapeutic concentration (Ostrovskaya et al., 2003). The detected phenomenon deserves a broad and comprehensive study of the structural and biological effects of dilute aqueous solutions of Dox.

Investigating the effect that diluted BAS systems have on the growth and development of hydrobionts is a convenient method for assessing their bioeffects. (Tushmalova et al., 2014; Mathias et al., 2018). Nowadays, high demand is shown for ecotoxicological tests, as they are closely connected with the pressing issue of protection of aquatic ecosystems from pharmaceutical products. It has been found that at low environmentally significant concentrations ranging from 10^6^ ng L^−1^ to 10^−2^ ng L^−1^, as well as at much lower calculated concentrations, some of the most dangerous pharma pollutants in the aquatic environment, such as antibiotics, nonsteroidal anti-inflammatory drugs, hormones can cause significant damage to hydrobionts (Santos et al., 2010; Ryzhkina et al., 2020; Chow et al., 2021; Wilkinson et al., 2022).

Dox has remained the most commonly used antitumor drug in recent decades (Lenz et al., 2007; Mahnik et al., 2007; Rivankar, 2014; Guven et al., 2018; Prasanna et al., 2020). It is known that anthracycline antibiotics, including Dox, are only partially metabolized, with Dox metabolites such as doxorubicinol also being toxic compounds (Lenz et al., 2007; Mahnik et al., 2007; Terekhova et al., 2019). It is estimated that 4–10% of Dox administered to a patient is excreted unmetabolized in urine (Lenz et al., 2007; Mahnik et al., 2007; Safarova et al., 2016; Terekhova et al., 2019). The main source of water body contamination with antibiotics, including cytostatics, is untreated waste in effluent from hospital complexes and pharmaceutical plants (Lenz et al., 2007; Mahnik et al., 2007; Terekhova et al., 2019). Analysis of wastewater from an oncology hospital by reversed-phase high performance liquid chromatography (Mahnik et al., 2007) showed that the concentration of Dox in hospital wastewater lies in the range of 1.10^2^–5.10^2^ ng L^−1^, which falls within the range of low environmentally significant concentrations of pharma-pollutants. In this regard, Dox systems at low calculated concentrations may serve as a convenient model for physicochemical investigation of the effects of low concentrations of antibiotics on hydrobionts, which are common and dangerous pollutants in surface waters.

The aim of this work is to study the self-organization, physicochemical properties, UV absorption and fluorescence of aqueous Dox systems in the range of calculated concentrations from 1·10^−20^ to 1·10^−4^ M, to predict and experimentally test their effects on representatives of hydrobionts and higher plants, establish the relationship between the non-monotonic concentration dependences of the disperse phase size, specific electrical conductivity, surface tension, pH, optical density, fluorescence intensity and bioeffects of these systems.

## Materials and methods

### Chemicals

Doxorubicin hydrochloride (Dox) was purchased from Sigma–Aldrich (Pharmaceutical Secondary Standard, United States). Solutions were prepared in 15 ml Wiegand (120011543) vials using only freshly prepared double-distilled water with specific conductivity of no more than 1.5 μS/cm and free of any particles, as checked by a Malvern Instruments Zetasizer Nano ZSP analyzer.

### Experimental design

The initial substrate’s solution of 1·10^−3^ M concentration was prepared from Doxdiluted with double-distilled water. Sample solutions (10 ml) were prepared *via* sequential decimal dilution, stirred using a minishaker (Shaker lab dancer, IKA, Staufen, Germany) for 10 s after dilution and kept for 20 h on the laboratory bench.

In the case of high dilutions (less than 1·10^−12^ M), the actual concentration of the substance is rather difficult to confirm by experimental methods. Therefore, in our previous papers (Konovalov and Ryzhkina, 2014; Konovalov A. et al., 2015; Ryzhkina et al., 2021a), as well as in the present work, when discussing high dilutions corresponding to calculated concentrations from 1·10^−13^ M to 1·10^−20^ M, we use this very term implying the theoretically possible concentration of the substance at a corresponding dilution step.

The method of analysis of highly diluted solutions involves the study of self-organization and properties of three parallel samples of the same concentration in two series (Konovalov A. et al., 2015). The difference between the first and the second series is that in the first one the samples are kept on the laboratory bench under ambient conditions before being studied by the physicochemical methods, and in the second they are kept in a cylindrical three-layer permalloy container that protects the contents from external electromagnetic fields (EMFs) with screening coefficients of ∼1000 (hypomagnetic conditions). Using this method, it is possible to establish a threshold concentration (c_thr_), below which structures formed in solutions are called nanoassociates, and above which are called domains (Konovalov and Ryzhkina, 2014; Konovalov A. et al., 2015). Starting from these concentrations and below, the DLS method cannot detect particles in solutions kept in the container.

Prior to the measurements, the solutions were kept at a constant temperature of 25 ± 0.1°C for 1 h (Konovalov and Ryzhkina, 2014).

## Physicochemical methods

### Conductometry

Changes in the specific conductivity (χ) of the solutions at 25 ± 0.1°C were determined using a conductometer (inoLab Cond Level 1, WTW, Weilheim, Germany).

### pH

pH was measured by a pH-meter (inoLab pH 720, WTW, Weilheim, Germany) at 25 ± 0.1°C.

### Tensiometry

Surface tension (σ) of the solutions at 25 ± 0.1°C was determined using a highly sensitive tensiometer (Sigma 720 ET, KSV Instruments, Helsinki, Finland).

### Dynamic light scattering

The particle size (the effective hydrodynamic diameter (*d*) of kinetically labile particles at the maximum of the distribution curve) was determined on a Zetasizer Nano ZSP analyzer (Malvern Instruments, Malvern, Worcestershire, UK) equipped with a 633 nm He–Ne laser and operating at an angle of 173°. The optical configuration using a 173° detection angle makes it possible to measure samples with low concentrations and particle sizes in the range from 0.6 nm to 6000 nm. The data were collected and analyzed using the Dispersion Technology Software version 7.10 (Malvern). The combination of these factors results in the exceptional sensitivity of the Zetasizer Nano ZSP analyzer, which is essential for the measurement of small nanoparticle sizes at low concentrations. In the case of diluted open self-organized systems, such as the studied Dox systems, the practical detection limit of the Zetasizer Nano ZSP analyzer is determined by the type of correlation function and polydispersity index (PI) automatically provided by the analyzer to the user. Each sample was measured in single-use polystyrene cuvettes (Sarstedt, Germany) with a pathlength of 10 mm. The measurements were made at a position of 4.65 mm distance from the cuvette wall with an automatic attenuator and at a controlled temperature of 25 ± 0.1°C.

### Electrophoretic light scattering

The ζ-potential of the Dox systems was determined on a Zetasizer Nano ZSP analyzer (Malvern Instruments Ltd., Malvern, Worcestershire, United Kingdom). Each sample was measured in a single-use U-shaped capillary cell (DTS 1071, Malvern Instruments Ltd., Malvern, Worcestershire, United Kingdom) at a controlled temperature of 25 ± 0.1°C. During the DLS and ELS methods investigations, the solutions were freed of dust by filtering through Iso-Disc N-25-4 Nylon (Supelco, Bellefonte, PA, United States) filters. Each point shown in concentration dependencies of size and ζ-potential is a result of statistical processing of data obtained by measuring the size or ζ-potential of three parallel samples of the same concentration. Each sample was measured six times. Then we used Microsoft Excel to find the arithmetic mean (*n* = 18) of the size or ζ-potential measurement and the standard deviation of the measurement. Concentration dependences show the arithmetic mean with the standard deviation for each measurement.

Statistical processing of the results was carried out by parametric statistics using Microsoft Excel with a statistical reliability of 95%. The measurement errors of nanoassociate parameters and physicochemical properties of solutions (specific electrical conductivity, pH, surface tension) were in the range of 2–20%.

### Scanning probe microscopy

The Dox system at a concentration of 1·10^−11^ M was examined using a Dimension Fast Scan scanning probe microscope (Bruker, United States). A drop of the sample was applied to the surface of a glass substrate and dried at room temperature until completely dry. SPM studies were performed in Quantative Nanomechanical Mapping mode using a Scan Asyst Fluid+ (Bruker, United States) probe (nominal length 70 μm, curvature radius 20 nm, stiffness 0.7 N/m). The probe clamping force was ∼2 nN, scanning speed was 0.977 Hz. The obtained data were processed using Nanoscope Analysis v.1.5 software.

### Scanning electron microscopy

Electron-microscopic analysis of the Dox system at a concentration of 1·10^−11^ M was performed using a MERLIN high-resolution scanning electron microscope with field emission (Carl Zeiss, Germany). The surface morphology was observed at an incident electron accelerating voltage of 5 kV and a cathode-ray current of 300 pA. The sample was sputtered on the holder in a Quorum vacuum chamber apparatus (Q 150T ES). The conductive layer was applied by cathodic sputtering with Au/Pd alloy (80/20). The thickness of the alloy was 15 nm. SPM and SEM studies were performed at the Interdisciplinary Center for Analytical Microscopy of Kazan Federal University.

### UV–vis spectroscopy

The UV absorption spectra for the Dox systems in the range of calculated concentrations from 1·10^−20^ M to 1·10^−4^ M were obtained using a UV/Vis Cary 100 spectrophotometer (Agilent Technologies, United States). This is a double beam spectrophotometer with Czerny–Turner monochromator, a wavelength range of 190–900 nm and a resolution of 0.1 nm. The accuracy and reproducibility of wavelengths were < ±0.02 and < 0.008 nm, respectively; the scanning rate was equal to 600 nm/min with an interval of 1 nm. We used QS-SUPRASIL quartz cells 10 mm in length. The UV absorption spectra of the Dox systems, obtained by excluding the baseline of the water used to prepare the solutions, were reproduced many times; the differences in the spectra of parallel-prepared samples were insignificant. The obtained data were analyzed using Microsoft Excel and Origin Pro 2015 software.

### Fluorescence spectroscopy

Fluorescence spectra were recorded using a Cary Eclipse Fluorescence Spectrophotometer (Agilent Technologies, United States). It is equipped with a xenon lamp and two Czerny–Turner monochromators. The slits on the excitation and emission monochromators had widths of 5 nm. The detector voltage was high. Standard (10 mm) quartz fluorescence cells (Part No. 6610000900, Agilent Technologies, Germany) were used. After their loading, we enabled a 10 min thermal equilibration. The cells were thermostated using a Peltier element. The emission and excitation spectra of Dox systems in the concentration range from 1·10^−4^ M to 1·10^−20^ M were obtained with the high voltage detector setting. We studied fluorescence spectra at excitation wavelengths (λ_ex_) of 225 nm, as well as excitation spectra at an emission wavelength (λ_em_) of 340 nm. The concentration dependencies of fluorescence intensity show the standard deviation obtained by processing the intensity (λ_em_ 340 nm) from three measurements of the same sample. The discrepancy between the results of the three parallel experiments did not exceed 20%. In cases where the shape of the fluorescence band changed, the given values represent an estimate. The obtained data were analyzed using Microsoft Excel and Origin Pro 2015 software.

### Toxicological methods

The toxic action of the Dox systems was tested using the certified procedures for the monitoring of toxicity of natural waters and wastewater.

### Biotesting on cladocerans *Ceriodaphnia affinis*

The method is based on the determination of the mortality and changes in fertility of *Ceriodaphnia affinis* when exposed to toxic substances present in the test solution, compared with the control culture in samples that do not contain toxic substances (control). The determination of the toxicity of each sample without dilution and each dilution is carried out in ten glasses in 2 parallel experiments and accompanied by a series of controls in 10 glasses. The temperature of the ambient air in the laboratory room is determined by the system of general conditioning of the laboratory premises and is equal to (22 ± 2)°C. An additional heating element is installed in the luminostat, which maintains (regulates) the temperature in the range (23 ± 1)°C. Biotesting is carried out in chemical glasses with a capacity of 30 ml, which are filled with 15 ml of the test solution, and where a single ceriodaphnia of no more than 24 h of age is placed. Cladocerans placing starts from the control series. Accounting the mortality of ceriodaphnia in the test and control samples is carried out within 48 h.

The percentage of dead ceriodaphnia was calculated by Eq. 1:

$$B=\frac{X_{c}-X_{t}}{X_{c}}\times 100\%$$ (1)

where, X_c_ and X_t_ are the number of survived individuals in control and in the test solution, respectively.

Toxicity of Dox solutions was determined from death rate of the cladocerans (*Ceriodaphnia affinis*). Acute toxicity was defined as death rate of at least 50% of the cladocerans during 48 h, provided that the death rate for the reference sample did not exceed 10%. Concentration of Dox was considered harmless (not leading to acute toxic effect) if the death rate did not exceed 10%.

### Biotesting on the wheat seeds of *Triticum vulgare*

The technique was based on measuring the length of the roots of wheat seedlings in the early stages of development. 25 dry disease-free seeds with germination of at least 95% were placed into a Petri dish. Then 5 ml of the test solution was added to each, and distilled water was added to the control Petri dish. All samples were placed in a thermostat for 7 days. Three parallel experiments were then conducted. After the experiment, the length of the longest roots was determined. The phytotoxic effect (%) was determined by comparing the length (*L*_*av*_, mm) of the roots of the control and experimental seeds Eq. 2:

$$L_{av}=\frac{\sum L_{i}}{n}$$ (2)

where, *Li* (mm) is the length of the maximal root of each seed, and n is the total number of seeds used for the experiment. Phytotoxic action was considered confirmed if the change in the root’s growth was at least 20%.

### Biotesting on *Chlorella vulgaris* green algae

The method was based on registering differences in the optical density of the test culture of the *C. vulgaris* green algae grown on a medium that did not contain toxic substances (control) and in the studied solutions.

2 ml of the test culture in 50% Tamiya medium containing 12.5 × 10^6^ cells/ml was introduced into 48 ml glass vessels containing control and test solutions. The contents of each glass were mixed and 6 ml of the mixture was then poured in 4 reactors, which were then placed in a multi-cell cultivator KVM-05 (T = (36 ± 0.5)°C, light intensity 60 W/m^2^, hold-up time 22 h). After 22 h, the optical density (λ = 560 nm) was measured in each reactor.

The relative difference in the average optical density (*l*, %) for each dilution compared with the control was calculated by Eq. 3:

$$l=\frac{D_{c}-D_{t}}{D_{c}}\times 100\%$$ (3)

where, D_c_ and D_t_ are average values of optical density in the control and test solution, respectively.

The toxicity criterion on algae tests was at least 20% change during 22 h in the optical density level due to inhibition or stimulation of the algae sample with respect to the reference.

## Results and discussion

**FIGURE 1:**
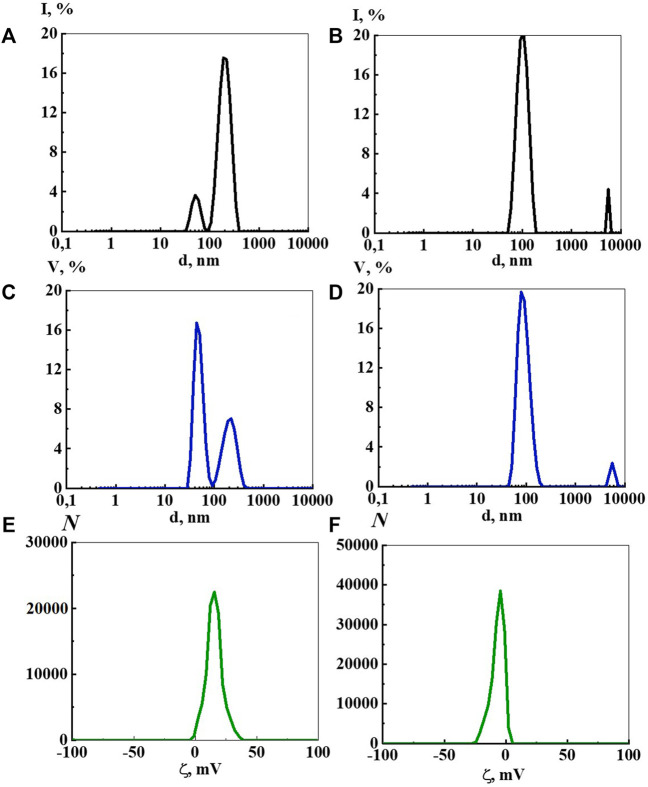
Particle size distribution based on light scattering intensity **(A,B)** and volume **(C,D)**, as well as ζ-potential **(E,F)** in the aqueous Dox systems at a concentration of 1·10^−4^ М **(A,C,E)** and 1·10^−9^ М **(B,D,F)**. Measurements were performed at 25 ± 0.1°C.

The DLS study of aqueous Dox solutions in a wide range of calculated concentrations from 1∙10^−20^ to 1∙10^−4^ M has shown that they are complex self-organized dispersed systems in which a dispersed phase of different nature forms upon dilution (Figures 1A–D), similarly to many BAS systems described earlier (Konovalov and Ryzhkina, 2014).

The bimodal distribution of light scattering intensity at a concentration of 1∙10^−4^ M (Figure 1A) indicates that the system contains micelle-like particles around 20–50 nm in size, formed mainly by molecules of a solute (Konovalov and Ryzhkina, 2014), and molecular-water domains hundreds of nm (about 200 nm) in size, formed by solute and ordered water structures (Konovalov and Ryzhkina, 2014; Kononov, 2015; Sed’lak, 2006; Sed’lak and Rak, 2013). Particle size distribution by volume (Figure 1C) confirms the bimodality of the disperse phase and its size, however, in contrast to the intensity distribution it indicates the predominant formation of micelle-like particles in this system. Arguments in favor of a micelle-like particle structure at 1·10^−4^ М are the amphiphilic nature of Dox and the positive ζ-potential value shown by ELS, which is characteristic of cationic surfactant micelles, as well as a small but reliably detectable decrease in the surface tension of the systems.

As a rule, as the systems dilute, the micelle-like particles transform into domains, and below a threshold concentration (*c*_thr_)—into nanoassociates formed mainly by ordered structures of water (Andrievsky et al., 2009; Zhernovkov et al., 2010; Belov et al., 2011; Pollack, 2013; Pershin et al., 2016; Yinnon, 2020).

In a number of works (Menozzi et al., 1984; Agrawal et al., 2009; Anand et al., 2012) complex spectral methods (circular dichroism, UV-vis absorption, fluorescence, nuclear magnetic resonance, mass spectrometry, molecular dynamics) were used to established that at concentrations in the range from 10^−3^ to 10^−6^ M Dox molecules have a strong tendency to self-association through stacking of aromatic chromophores and formation of stacked dimers and/or higher order aggregates in aqueous solutions. A transmission electron microscopy study of the substrate morphology of aqueous Dox solution at a concentration of 5∙10^−1^ mg/ml showed the presence in solution of internally non-uniform particles of round shape about 50 nm in size (Fülöp et al., 2013), which agrees well with our results.

At Dox concentrations of 1∙10^−5^ and 1∙10^−6^ M particles are not detected by the DLS method, probably due to a significant decrease in the number of Dox aggregates and system rearrangement. Indeed, from 1∙10^−7^ M and below, the size distribution by intensity (Figure 1B) and volume (Figure 1D) mainly indicates the formation of a dispersed phase of hundreds of nanometers in size (domains and nanoassociates), whose nature can be established similarly to (Konovalov and Ryzhkina, 2014; Konovalov A. et al., 2015), using a permalloy container shielding low-frequency EMF.

Holding solutions in a permalloy container shows that the threshold concentrations (*c*_thr_), which separate concentration regions where domains and nanoassociates form, for Dox systems are 1∙10^−9^, 1∙10^−8^ M (see Materials and Methods, Experimental Design). Concentration ranges close to c_thr_ are often characterized by significant changes in the properties of self-organized systems (Konovalov and Ryzhkina, 2014). Thus, the range of low concentrations of Dox systems can be indicatively divided into three intervals in which mainly micelle-like particles (1∙10^−4^ M), domains (1∙10^−8^, 1∙10^−7^ M), and nanoassociates (1∙10^−20^–1∙10^−9^ M) are formed.

An ELS study of the Dox systems found that the ζ-potential of micelle-like particles at 1∙10^−4^ M is +17.0 mV (Figure 1E), while for domains at 1∙10^−8^ and 1∙10^−7^ M it is −6.2 mV (Supplementary Figure S1), and for nanoassociates in the range from 1∙10^−11^ to 1∙10^−9^ M it changes non-monotonically from −3.5 mV to −12.0 mV (Figure 1F). At lower Dox concentrations, the data on the ζ-potential of nanoassociates are inadequate. The positive ζ-potential value of micelle-like particles at 1∙10^−4^ M can probably be explained by the fact that the work studies doxorubicin hydrochloride that has a charged ammonium group which promotes the formation of positively-charged micelle-like particles. The negative sign of the ζ-potential of domains and nanoassociates, according to (Andrievsky et al., 2009; Pollack, 2013; Yinnon, 2020), may be related to the properties of ordered water structures involved in the formation of a dispersed phase of hundreds of nm in size.

**FIGURE 2:**
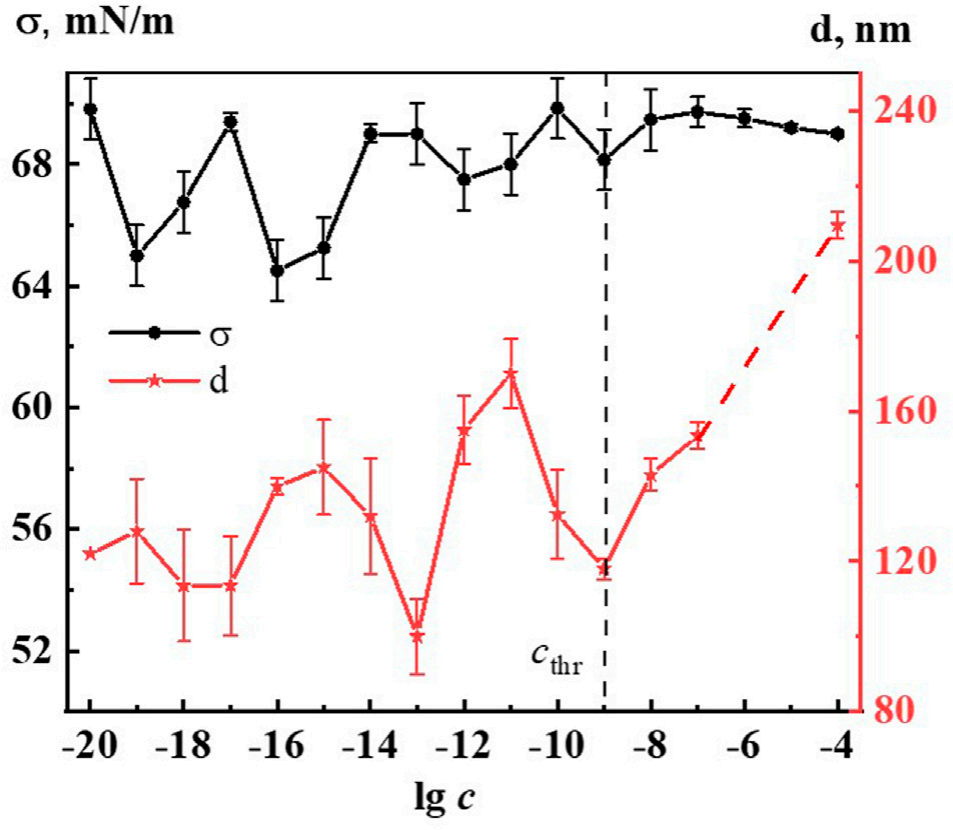
Dependence of particle size (d) and surface tension (σ) on concentration (c, M) of the Dox systems. Measurements were performed at 25 ± 0.1°C. The dotted line indicates the threshold concentration (*c*_thr_).

Figure 2 shows the nonmonotonic concentration dependence of disperse phase size in hundreds of nm, indicating *c*_thr_, which shows that the increase in nanoassociates size is observed in three regions: 1∙10^−19^, 1∙10^−16^–1∙10^−15^, 1∙10^−12^–1∙10^−11^ M.

An important characteristic of a dispersed system is the polydispersity index (PI): the smaller its value, the greater the dimensional homogeneity of the dispersed phase, the more ordered the system is (Bhattacharjee, 2016).

Analysis of the PI of the Dox systems showed that over the entire range of studied concentrations, the PI is approximately 0.30 except for concentrations of 1∙10^−9^ M (*c*_thr_) and 1∙10^−15^ M, at which it is 0.38 and 0.22, respectively, which may be related to the coherent change in the properties of the systems at these concentrations.

**FIGURE 3:**
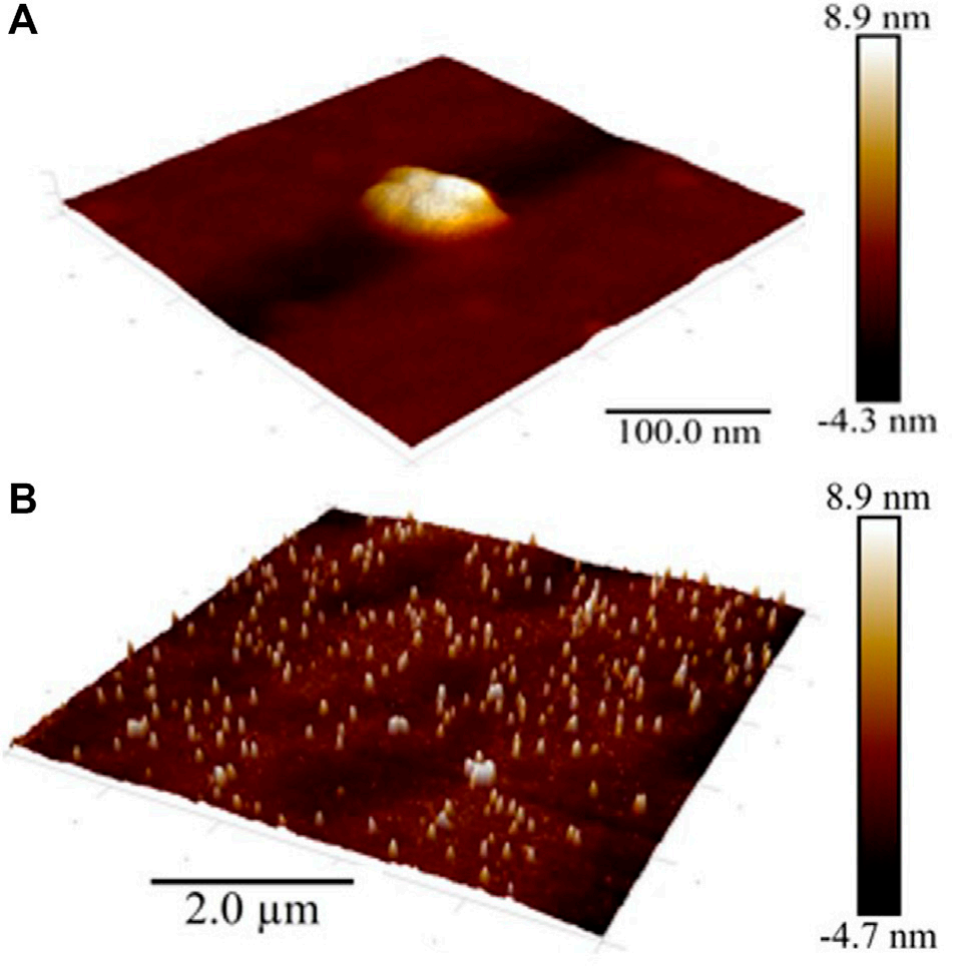
SPM images of the surface of the Dox aqueous system at 1·10^−11^ M Scale bar: **(A)** 100 nm, **(B)** 2 µm.

**FIGURE 4:**
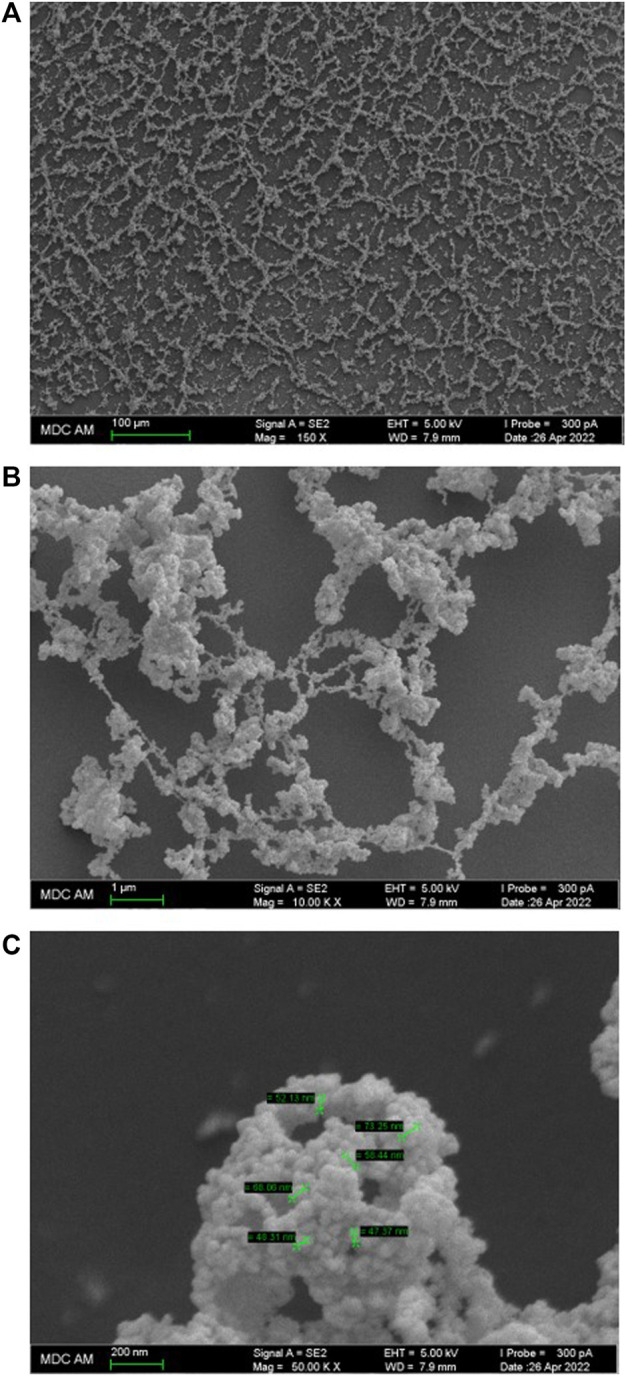
SEM images of the surface of the Dox aqueous system at 1·10^−11^ M. Scale bar: **(A)** 100 μm, **(B)** 1 μm, **(C)** 200 nm.

Atomic force microscopy (AFM) methods, including scanning probe microscopy (SPM) and scanning electron microscopy (SEM) are successfully used to visualize and reveal the features of the dispersed phase formed in dilute aqueous BAS systems (Konovalov and Ryzhkina, 2014; Padnya et al., 2017; Ryzhkina et al., 2017). We chose to use SPM (Figure 3) and SEM (Figure 4) methods to study the Dox solution with a concentration of 1∙10^−11^ M, which is a dispersed system in which negatively charged nanoassociates of hundreds of nm in size are formed (Figures 1B,D,F), which are analogous to soft nanomaterials such as nanogels (Kabanov and Vinogradov, 2009; Lo et al., 2009; Lo et al., 2012; Ho, 2014; Ho, 2015), whose properties are widely studied using SPM and SEM (Gonsalves et al., 2007; Garcia, 2020).

As can be seen from Figures 3A,B, as well as Supplementary Figures S2A,B (see Supplementary Material), the entire substrate is covered with a thin fine-grained film with an average roughness of ∼1.5 nm (grain height ∼ 1.5 nm, diameter ∼ 2.5 nm) bearing soft particles typical of soft materials (Hobbs et al., 2009; Lo et al., 2012; Ho, 2015; Garcia, 2020) with an average diameter of about 100 nm (50–200 nm) and a height of 10–14 nm, formed by 2-8 closely spaced, smaller particles with an average base diameter of about 15–20 nm. The particles are located 50–500 nm a part, forming chains that are twisted into spatially oriented structures in the form of intertwined rings and spirals of 2–5 μm. As a control sample, we examined freshly prepared double-distilled water kept in vial for days. The surface of a water sample is covered by a very thin film with an average roughness of ∼0.1 nm, on which there are almost no particles (Supplementary Figure S3A). The height of the film and single particles on the surface of a water sample is significantly lower (more than by an order of magnitude) compared to the sample of a Dox aqueous system at 1·10^−11^ M. Obtained data are in good agreement with those reported by (Elia et al., 2013), who showed that the height of the clusters of iteratively nafionated water is much higher compared to the reference sample of pure water.

A similar thin film with a chain orientation of its constituent soft particles is shown in AFM images obtained in intermittent contact mode (tapping-mode) when studying aqueous systems based on amphiphilic calix [4] resorcinarene and (*S*)-Lys in the range of low concentrations in which the DLS method shows the formation of nanoassociates mainly consisting of structured water molecules (Konovalov and Ryzhkina, 2014; Ryzhkina et al., 2017). As established earlier, the film is an ordered water of the EZ-type that doesn’t dry on the substrate (Lo et al., 2012; Elia et al., 2013; Ho, 2015; Yinnon et al., 2016).

The SPM data are in good agreement with the SEM study of the Dox solution. Figures 4A,B,C show that the substrate is covered with a branched network formed by interconnecting chains 50–200 nm thick, twisted into rings, spirals, and webs ranging in size from tens to several hundred micrometers. The chains, in turn, are formed by thin filaments composed of tightly adhering particles of ∼20–50 nm in diameter (Figure 4B). The chains and filaments are folded into a variety of fine particles hundreds of nm in size, woven into an overall branched network (Figure 4C).

Works that jointly use DLS and SEM methods (Padnya et al., 2017) show that data obtained by these methods are in good agreement with each other. In our work, the data obtained by DLS and SEM methods also confirm and complement each other. The DLS method shows bimodal particle size distribution in aqueous Dox systems with the formation of dispersed phase of hundreds and thousands of nm in size (Figure 1), which can be seen in SEM images. At the same time, the SEM method visualizes the discreteness of the dispersed phase, probably achieved due to the ζ-potential, and its fractal nature, shown in (Lunev et al., 2014), which causes self-assembly into spatially organized structures on the substrate.

Consequently, the SPM and SEM results confirm the DLS data on the formation of a dispersed phase of hundreds of nm in size in dilute Dox solutions, consisting mainly of ordered water, and also indicate its ability, similar to other soft materials (Lo et al., 2012; Elia et al., 2022), to self-assemble into spatially organized structures on a substrate.

It is established that nanoassociates’ formation and rearrangement causes the emergence of nonmonotonic concentration dependences of physicochemical properties of the systems (Konovalov and Ryzhkina, 2014). Figure 2 shows non-monotone interrelated concentration dependences of nanoassociates size (*d*) and surface tension (σ), and Supplementary Figures S4–S6 (see Supplementary Material) show dependences of *d* and specific conductivity (χ), as well as *d* and pH, and also σ and χ of Dox systems.

All figures clearly show the relationship of the above dependencies, which below *c*_thr_ change in counter-phase (*d* and σ) or in-phase (*d* and χ, *d* and pH) with extremums in the vicinity of certain critical concentrations 1∙10^−19^, 1∙10^−16^ −1∙10^−15^, 1∙10^−12^–1∙10^−11^ M. A similar course of the dependences of *d* and σ, as well as of *d* and χ was shown in work (Ryzhkina et al., 2022) that studied aqueous systems of (*L*)-Tryptophan, whose molecules are amphiphilic similarly to Dox, and whose aqueous systems are capable of forming nanoassociates. Coherent changes in *d, σ*, χ, and pH are also observed in the vicinity of a threshold concentration of 1∙10^−9^M of the Dox systems (Figure 2, Supplementary Figures S4–S6). As shown in (Konovalov A. et al., 2015; Ryzhkina et al., 2021c; Ryzhkina et al., 2022), the local decrease of the surface tension at certain low concentrations, which is responsible for the membranotropic properties of disperse systems, indicates the possibility of more pronounced bioeffects at these concentrations of BAS, which for Dox systems will be discussed in more detail below.

Thus, the rearrangement of nanoassociates in Dox systems is accompanied by a consistent nonmonotonic change in the physicochemical properties - surface tension, specific conductivity, and pH, with extremes at the same critical concentrations.

**FIGURE 5:**
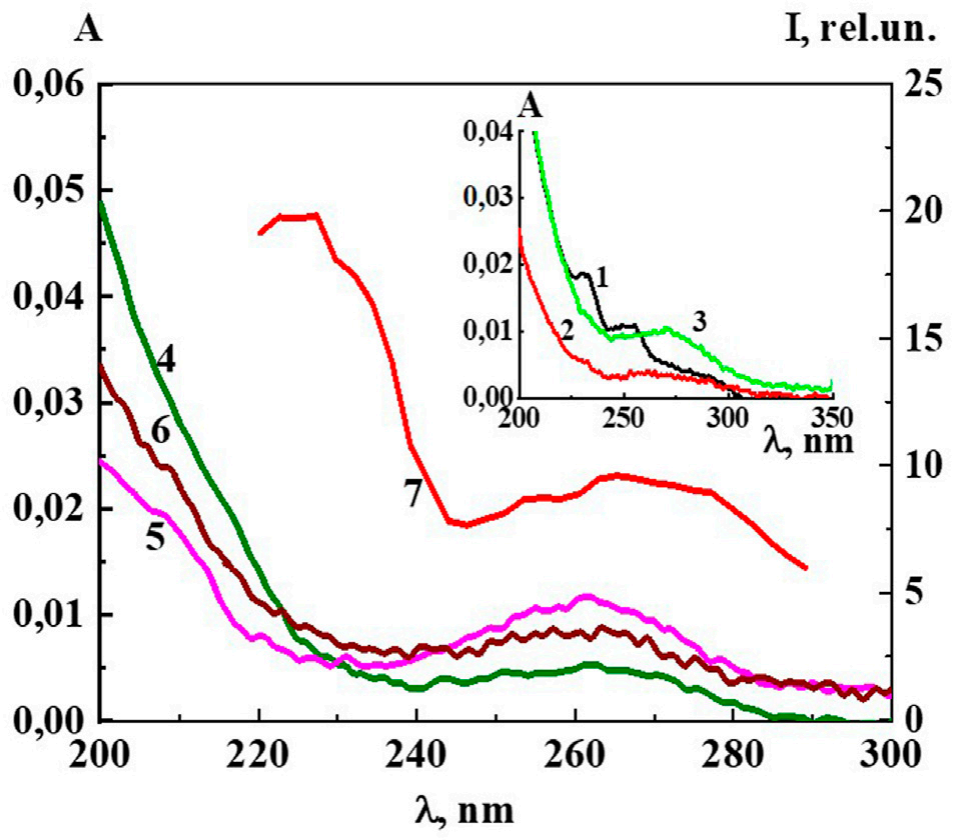
The absorption spectra of the Dox systems at 1) 1·10^−6^ M (Insert), 2) 1·10^−7^ M (Insert), 3) 1·10^−8^ M (Insert), 4) 1·10^−13^ M, 5) 1·10^−16^ M, 6) 1·10^−19^ M and 7) the excitation spectra (λ_em_340 nm) of the Dox system at 1·10^−16^ M. Insert: The absorption spectra of the Dox systems at 1) 1·10^−6^ M, 2) 1·10^−7^ M, 3) 1·10^−8^ M. Measurements were performed at 25 ± 0.1°C.

UV absorption spectra (A) of Dox systems between 1∙10^−20^ M and 1∙10^−6^ M, obtained by subtracting the baseline of the water used to prepare the solutions, are shown in Figure 5 and Insert. The UV spectrum of the system with a concentration of 1∙10^−6^ M shows bands 288, 252, 233 nm (Figure 5, Insert), typical of Dox systems in the concentration range 1∙10^−5^ −1∙10^−4^ M (Agrawal et al., 2009; Anand et al., 2012). Some authors believe that the bands at λ 288, 252, 233 nm belong to the aromatic ring and the daunosamine part of the Dox molecule (Agrawal et al., 2009). Starting at a concentration of 1·10^−7^ М and below, the spectra have a different appearance, typical of the previously described systems of BACs in similar low concentration intervals in which nanoassociates form, with very weak absorption in the 215–225 nm region and a somewhat more pronounced shoulder in the 260 nm region (Ryzhkina et al., 2021a; Ryzhkina et al., 2021b; Ryzhkina et al., 2021c; Ryzhkina et al., 2022).

Careful measurements of the UV spectra of the Dox systems show small but distinct differences in the absorption curves as they are being diluted (Figure 5). The nonmonotonic concentration dependences of A_225_ and A_260_ are highly correlated (Supplementary Figures S7), indicating that both bands are associated with structural rearrangements of the nanoassociates and concentration-dependent changes in system properties such as χ, similarly to (Ryzhkina et al., 2018; Ryzhkina et al., 2022).

**FIGURE 6:**
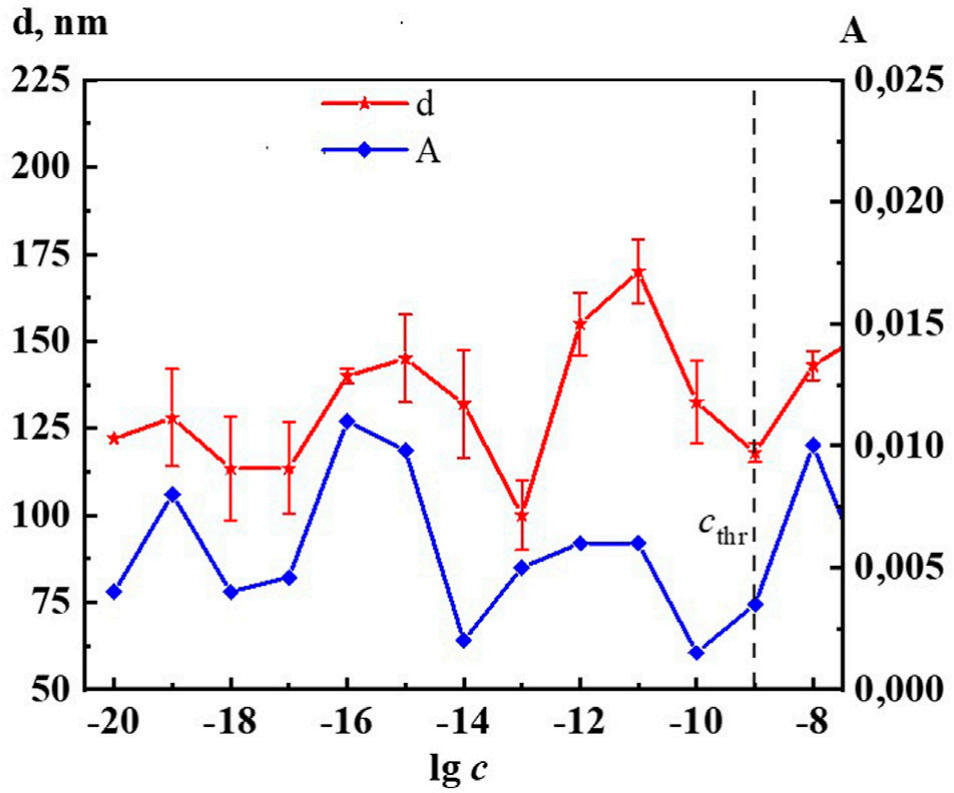
Dependence of particle size (d) and absorbance at λ 260 nm (A) on concentrations (c/M) of Dox. Measurements were performed at 25 ± 0.1°C. The dotted line indicates the threshold concentration (*c*_thr_).

**FIGURE 7:**
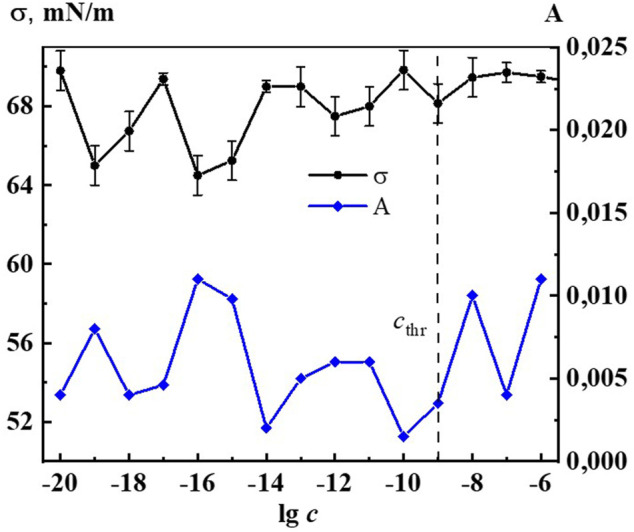
Dependence of surface tension (σ) and absorbance at λ 260 nm (A) on concentrations (c/M) of Dox. Measurements were performed at 25 ± 0.1°C. The dotted line indicates the threshold concentration (*c*_thr_).

Indeed, Figures 6, 7 and Supplementary Figure S8 show that the changes of A_260_, as well as *d,* σ and χ on the corresponding concentration dependences are nonmonotonic and coherent with extremes at critical concentrations of 1·10^−19^, 1·10^−16^–1·10^−15^, 1·10^−12^–1·10^−11^ М. The obtained data confirm the earlier conclusion that the UV band in aqueous dilute systems of BAS with a shoulder in the region of 215–230 nm and 260–270 nm is due to the formation of nanoassociates (Ryzhkina et al., 2018; Ryzhkina et al., 2022).

**FIGURE 8:**
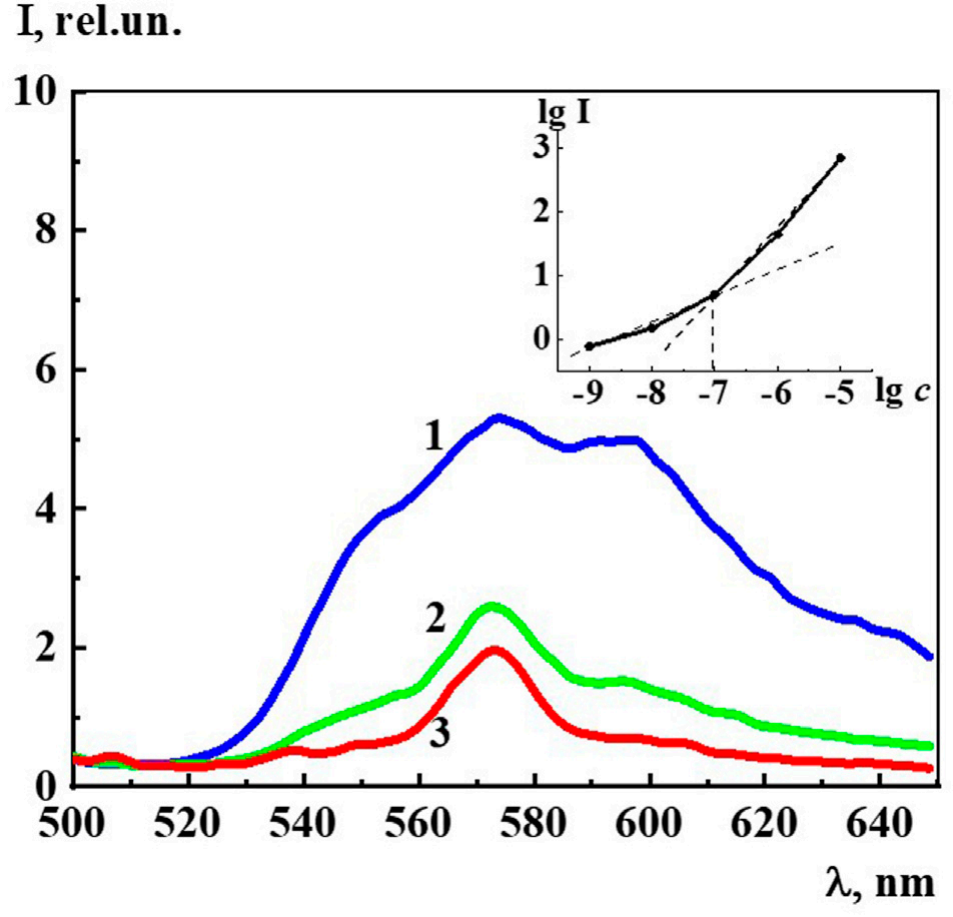
The fluorescence spectra (λ_ex_480 nm) of the Dox systems at 1) 1·10^−7^ M, 2) 1·10^−8^ M, 3) 1·10^−9^ M. Insert: Dependence of logarithm fluorescence intensity (λ_ex_480 nm, λ_em_590 nm) (lg I) on logarithm concentrations (c/M) of Dox.

Figure 8 shows the fluorescence spectra of an aqueous solution of Dox at concentrations of 1∙10^−7^ M, 1∙10^−8^ M, and 1∙10^−9^ M related to the monomeric form of Dox, since the dimer is known to be none missive at all (Agrawal et al., 2009). The spectra have a complex structure in which three main maxima with λ equal to 555 nm, 577 nm, and 590 nm are distinguished. Insert of Figure 8 shows the logarithmic dependence of the fluorescence intensity (λ_ex_ 480 nm, λ_em_ 590 nm) on the Dox concentration in the range 1∙10^−9^ to 1∙10^−5^ M, which has a kink at 1∙10^−7^ M. This may imply that, starting from this concentration and below, the system is rearranged, accompanied by a change in the polarity of the water molecules and the nature of their interaction with the Dox chromophore groups. Indeed, the DLS method shows that starting from 1∙10^−7^ M and below, a dispersed phase of hundreds of nm in size is formed in the Dox aqueous system (Figure 1) consisting of Dox molecules and quasi crystalline water of EZ-type, significantly different from the “bulk” (Andrievsky et al., 2009; Zhernovkov et al., 2010; Belov et al., 2011; Pollack, 2013; Pershin et al., 2016; Yinnon, 2020).

**FIGURE 9:**
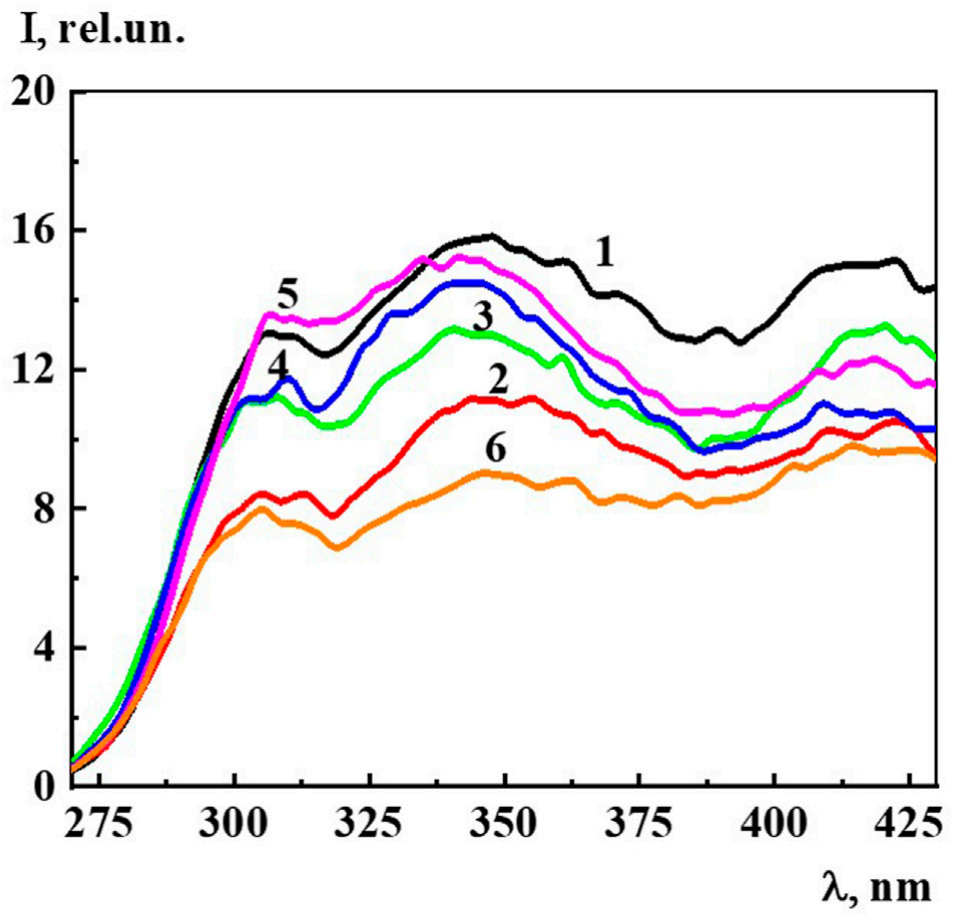
The fluorescence spectra (λ_ex_225 nm) of the Dox systems at 1) 1·10^−7^ M, 2) 1·10^−8^ M, 3) 1·10^−9^ M, 4) 1·10^−10^ M, 5) 1·10^−15^ M, 6) 1·10^−19^ M. Measurements were performed at 25 ± 0.1°C.

The fluorescence spectra (λ_ex_ 225 nm) of Dox systems in the concentration range from 1∙10^−19^ to 1∙10^−7^ M, in which domains and nanoassociates are formed (Figure 9), look similar to the previously described highly diluted BACs (Ryzhkina et al., 2021a; Ryzhkina et al., 2021b; Ryzhkina et al., 2021c; Ryzhkina et al., 2022). The fluorescence spectra show three broad overlapping bands in the spectral ranges 300–320 (band 310), 325–375 (band 340), and 400–440 (band 425) nm, whose shape does not change much with dilution.

**FIGURE 10:**
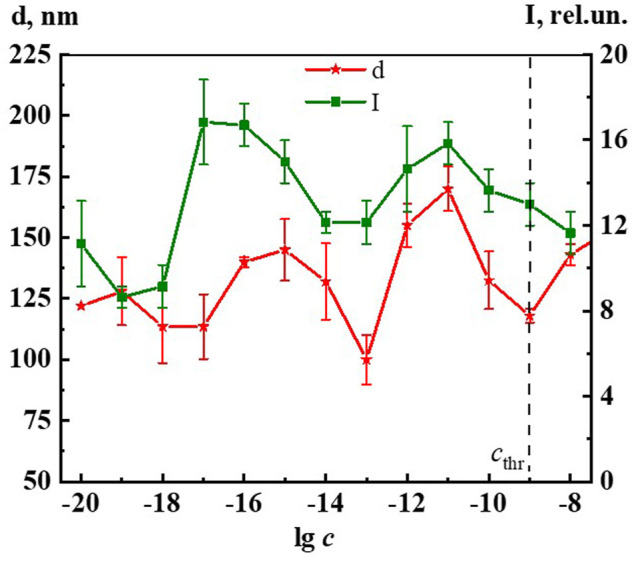
Dependence of particle size (d) and fluorescence intensity (λ_ex_225 nm, λ_em_340 nm) (I) on concentrations (c/M) of Dox. Measurements were performed at 25 ± 0.1°C. The dotted line indicates the threshold concentration (*c*_thr_).

Since, as in the case of A_225_ and A_260_, the intensity of the 310, 340, and 425 nm bands changes symbatically with dilution, we select the 340 band similarly to (Ryzhkina et al., 2021a; Ryzhkina et al., 2021b; Ryzhkina et al., 2021c; Ryzhkina et al., 2022) to estimate the fluorescence intensity (I) and plot the concentration dependence (Figure 10). The data obtained indicate that as the concentration decreases in the range 1∙10^−20^ to 1∙10^−8^ M, while the 340 nm band shape (λ_ex_ 225 nm) remains generally the same, there is a nonmonotonic change in its intensity, which according to (Ryzhkina et al., 2021a; Ryzhkina et al., 2021b; Ryzhkina et al., 2021c; Ryzhkina et al., 2022) suggests that the 340 band in Dox systems, just as in other highly diluted BACs solutions, is related to the formation and rearrangement of nanoassociates.

Figure 5 shows the excitation spectra (λ_em_ 340 nm) of the Dox system at a concentration of 1∙10^−16^ M (spectrum 5). The spectrum shows two slightly overlapping broad bands in the short-wave (215–240 nm) and long-wave region (250–290 nm). It can be seen that the short-wave band with a maximum at 220 nm is twice as intense as the long-wave band with a maximum at 270 nm. The excitation and absorption spectra are close, which is characteristic of fluorescent systems (Jameson, 2014). Excitation spectra of Dox systems are similar to the spectra of highly diluted BAS systems obtained earlier (Ryzhkina et al., 2021a; Ryzhkina et al., 2021b; Ryzhkina et al., 2022).

Figure 10 shows non-monotonic concentration dependences of the dispersed phase size (*d*) and fluorescence intensity (λ_ex_ 225 nm, λ_em_ 340 nm) (I) of Dox systems, indicating a consistent change in the nanoassociates size and their intrinsic ability to absorb and emit energy in the UV region in certain concentration intervals. Considering the established interconnection between the size of nanoassociates, physicochemical properties, UV absorption and fluorescence of the systems, we can conclude that the previously found (Ryzhkina et al., 2021a) pattern of the determining role of nanoassociate rearrangements in the occurrence of coherent nonmonotonic changes in the properties of disperse systems in the low concentration range is also true for the Dox systems.

In the framework of the previously expressed hypothesis (Konovalov and Ryzhkina, 2014; Ryzhkina et al., 2021a) it can be assumed that Dox systems are able to affect living systems in the range of low concentrations, in which a dispersed phase is formed and non-monotonic changes in physicochemical and spectral (fluorescence intensity) properties are observed.

The certified procedures for monitoring the toxicity of natural waters and wastewater (see Materials and methods, Toxicological methods) were used to check the possible harmful effects of diluted Dox solutions on hydrobionts and higher plants.

Testing the effect of Dox systems on *Triticum vulgare* wheat root growth in the range 1∙10^−19^ - 1∙10^−6^ M showed that at concentrations of 1∙10^−19^, 1∙10^−11^ and 1∙10^−6^ M a weak stimulating effect (increase in root length by 11–15% compared with control) was observed. At other concentrations, the Dox system has no effect on root growth. The sensitivity of the *Ceriodaphnia affinis* cladocerans and the *Chlorella vulgaris* unicellular green algae to the action of Dox systems was significantly higher.

The experiment to study the effect on cladoceran mortality revealed toxic effects (50–100% mortality) of Dox systems in the domain formation concentration interval of 1∙10^−9^–1∙10^−6^ M and nonmonotonic harmful effects (10–20% mortality) in the nanoassociate formation interval of 1∙10^−19^–1∙10^−11^ M with maxima at 1∙10^−19^, 1∙10^−15^, 1∙10^−11^ M.

**FIGURE 11:**
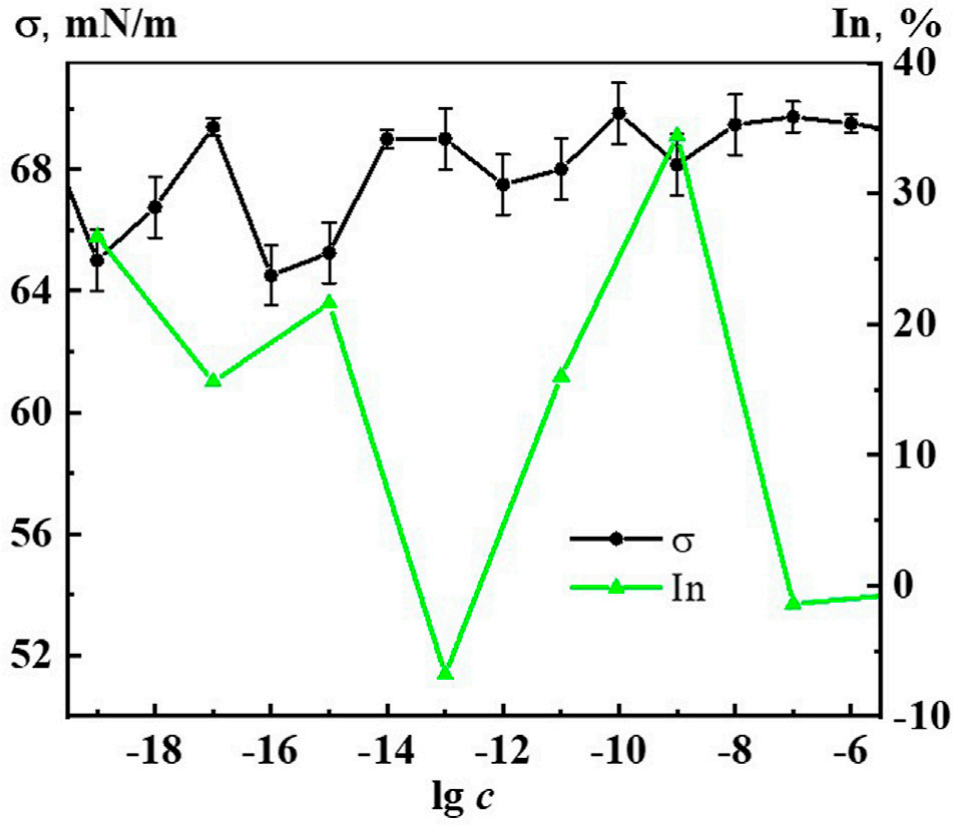
Dependence of surface tension (σ) and inhibition of the abundance of *Chlorella vulgaris* green algae (In) on concentration (c, M) of the Dox systems.

A study of the effect of Dox systems on the *Chlorella vulgaris* unicellular green algae showed (Figure 11) that at domain formation concentrations of 1∙10^−7^, 1∙10^−6^ M the systems have no effect on algal abundance. Starting from a threshold concentration of 1∙10^−9^ M and below, there is a non-monotonic inhibition of algal growth by the Dox systems, which reaches its highest value at 1∙10^−19^, 1∙10^−15^, 1∙10^−9^ M, being 27%, 22%, 35% compared to control, respectively. In the vicinity of 1·10^−13^ M the harmful effect of Dox systems on unicellular green algae is practically absent (silent zone).

When comparing the results of ecotoxicological experiments with data on the self-organization and properties of Dox systems, it is easy to notice that harmful effects on hydrobionts occur in the vicinity of critical concentrations 1·10^−19^–1·10^−20^, 1·10^−15^–1·10^−17^, 1·10^−11^–1·10^−12^ M, where the most significant changes in nanoassociate parameters, physicochemical properties, optical density, fluorescence intensity of the systems are observed (Figures 2, 6, 7, 10, 11 and Supplementary Figures S4–S6, S8, S9). At the same time, the degree of exposure and the sign of bioeffects depend on the properties of the disperse system at a given dilution, as well as to a large extent on the nature of the test objects.

It is known that the permeability of biomembranes increases when the surface tension at the interface decreases, reinforcing the bioavailability of substances forming the dispersed phase (Konovalov and Ryzhkina, 2014; Konovalov A. et al., 2015; Ryzhkina et al., 2021c; Ryzhkina et al., 2022). Figure 11 shows coherent non-monotonic concentration dependences of green algae abundance inhibition (In) under the effect of Dox systems and surface tension systems (σ). It is well seen that the peaks of the bioeffect and the reduction of σ occur in the vicinity of the abovementioned critical concentrations. This confirms the conclusion that the mechanism of the effect of Dox systems on bioobjects in the interval of nanoassociates formation similarly to the works (Konovalov and Ryzhkina, 2014; Konovalov A. et al., 2015; Ryzhkina et al., 2021c; Ryzhkina et al., 2022) may be associated with the modification of the biomembrane structure under the coordinated action of the disperse phase and the change in the properties of the medium.

The results obtained are consistent with the literature data concerning the sensitivity of experimental animal tumor models (Lewis lung carcinoma, Ehrlich carcinoma, L1210 leukemia, 755 adenocarcinoma, B-16 melanoma) to Dox solutions at calculated concentrations of 1·10^−20^, 1·10^−15^, 1·10^−10^, 1·10^−5^ M (Ostrovskaya et al., 2003). The study of the effects of diluted Dox solutions on the listed tumor strains revealed a non-monotonic character of the concentration dependence of the anti-tumor effect of Dox solutions, which in certain concentration ranges is comparable to the inhibitory activity of the drug at a therapeutic dose of 1.10^−3^ M. It was found that the severity and direction of the bioeffect (inhibition-stimulation of tumor growth) depends not only on the concentration of the drug solution, but also on the nature of the tumor model.

## Conclusion

Thus, it was shown that aqueous solutions of the highly effective cytostatic antibiotic Dox in the calculated concentration range 1∙10^−20^–1∙10^−4^ M are self-organized disperse systems in which domains and nanoassociates of hundreds of nanometers in size are respectively formed above and below the threshold concentration, capable of rearranging with changes in size and ζ-potential as the dilution occurs. This is followed by non-monotonic coherent changes in the physicochemical properties, the appearance of absorption in the 210–300 nm region and fluorescence (λ_ex_ 225 nm) of the systems in the UV spectrum. The SPM and SEM results confirm and complement the DLS and ELS data indicating the existence of nanoassociates sized hundreds of nm in dilute Dox solutions, which are capable of self-assembly into spatially organized structures on the substrate. For the first time, the relationship between nanoassociates size, physicochemical properties, optical density A_260_ and fluorescence intensity (λ_ex_ 225 nm, λ_em_ 340 nm) of Dox systems and harmful effects in the calculated concentration range 1∙10^−19^– 1∙10^−6^ M on the growth and development of animal and plant organisms was established. Concentrations in the vicinity of 1·10^−19^–1·10^−20^, 1·10^−15^–1·10^−17^, 1·10^−11^–1·10^−12^ M, where non-monotonic harmful effects on *Ceriodaphnia affinis* cladocerans*,* inhibition of growth of *Chlorella vulgaris* green algae cell numbers, and minor stimulation of *Triticum vulgare* wheat root initial growth are achieved, coincide with the critical concentrations at which the greatest change in the size of nanoassociates, physicochemical and spectral properties (fluorescence intensity) of the system is observed. The results obtained are in agreement with literature data concerning the sensitivity of experimental animal tumor models to Dox solutions at calculated concentrations of 1·10^−20^, 1·10^−15^, 1·10^−10^ M. Given the wide range of applications of Dox, establishing the ability of its solutions in the range of low calculated concentrations to self-organize could be the key to understanding the mechanism of action of Dox systems on biobjects and developing approaches to their more effective and safe use.

## Data Availability

The original contributions presented in the study are included in the article/Supplementary Material; further inquiries can be directed to the corresponding author.
