# Supplementary material for: Doxorubicin aqueous systems at low concentrations: Interconnection between self-organization, fluorescent and physicochemical properties, and action on hydrobionts
Source: Front Chem. 2022 Dec 1;10:1063278. doi: 10.3389/fchem.2022.1063278 (PMC9751371; doi:10.3389/fchem.2022.1063278)
Supplement: Supplementary file 1 [file DataSheet1.docx]

Supplementary Material

**Supplementary Figure S1.** ζ-Potential in the aqueous Dox systems at a concentration of 1∙10^-7^ М. Measurements were performed at 25 ± 0.1°C.

| A | 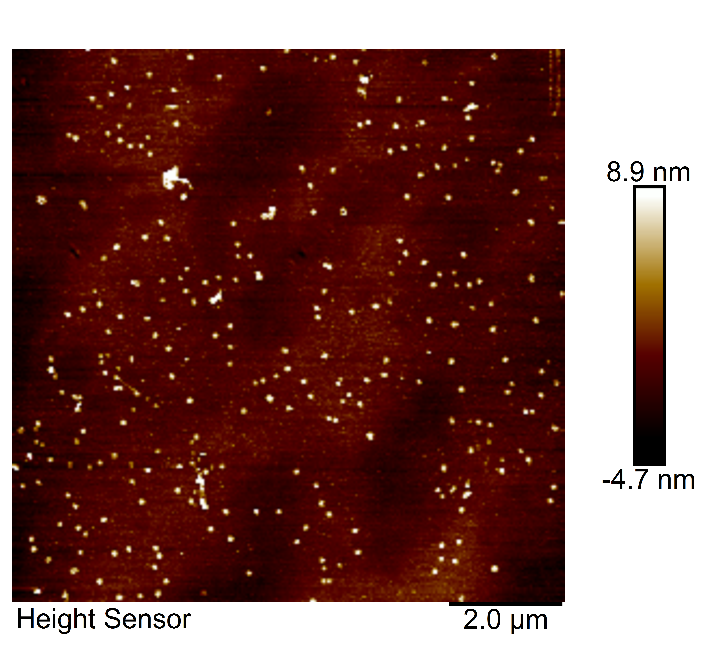 |
| --- | --- |
| B | 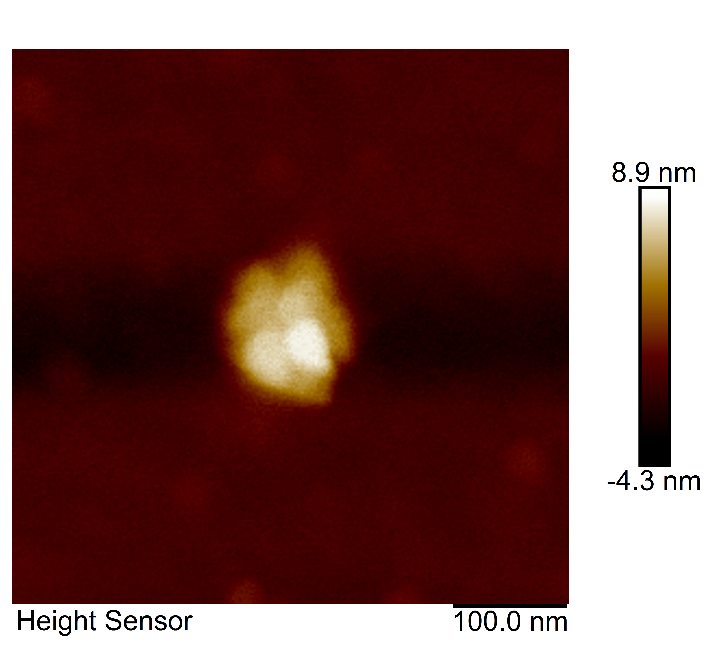 |

**Supplementary Figure S2.**SPM images of surface of the Dox aqueous system at 1·10^-11^ M.

Scale bar: A- 2.0 μm, B- 100.0 nm

| A | 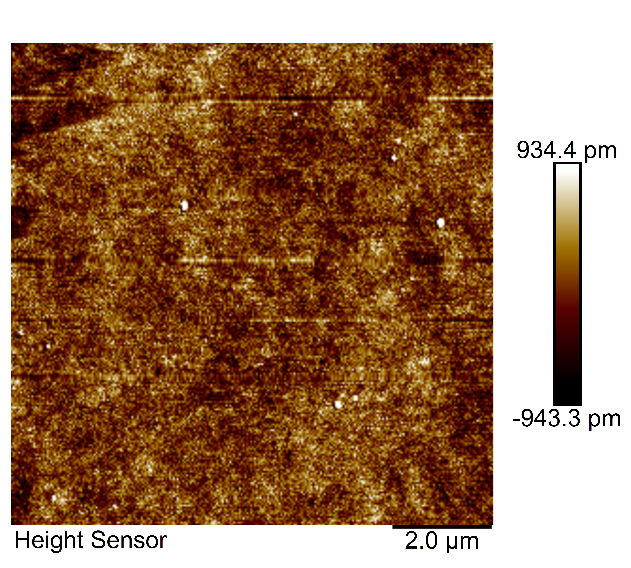 |
| --- | --- |
| B | 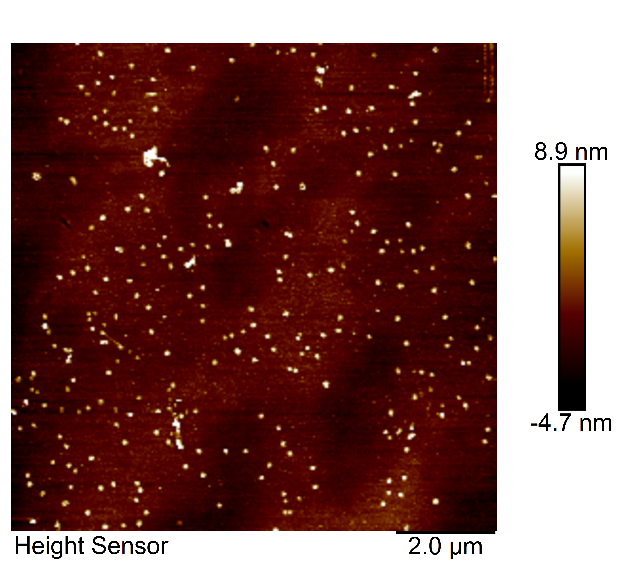 |

**Supplementary Figure S3.** SPM images of surface of a water control sample (A) and the Dox aqueous system at 1·10^-11^ M (B).Scale bar: A- 2.0 μm, B-2.0 μm.

**Supplementary Figure S4.** Dependence of particle size (d) and specific conductivity (χ) on concentration (c, M) of the Dox systems. Measurements were performed at 25 ± 0.1°C.

**Supplementary Figure S5.** Dependence of particle size (d) and pH on concentration (c, M) of the Dox systems. Measurements were performed at 25 ± 0.1°C.

**Supplementary Figure S6.** Dependence of surface tension (σ) and specific conductivity (χ) on concentration (c, M) of the Dox systems. Measurements were performed at 25 ± 0.1°C.

**Supplementary Figure S7.** Dependences of absorbance at 230 nm (1) and 260 nm (2) on concentrations (c/M) of Dox. Measurements were performed at 25 ± 0.1°C.

**Supplementary Figure S8.** Dependence of specific conductivity (χ) and surface tension (σ) and absorbance at 260 nm (A) on concentrations (c/M) of Dox. Measurements were performed at 25 ± 0.1°C.

**Supplementary Figure S9.** Dependence of surface tension (σ) and fluorescence intensity (λ_ex_230 nm, λ_em_340 nm) (I) on concentration (c, M) of the Dox systems. Measurements were performed at 25 ± 0.1°C.
